# SAFETY CLIMATE IN NURSING HOMES: ARE SENIOR MANAGERS IN AGREEMENT WITH DIRECT CARE STAFF?

**DOI:** 10.1093/geroni/igz038.242

**Published:** 2019-11-08

**Authors:** Emma Quach, Lewis Kazis, Shibei Zhao, Sarah McDannold, Christine W Hartmann

**Affiliations:** 1 VA Center for Healthcare Organization and Implementation Research, Bedford, Massachusetts, United States; 2 Boston University School of Public Health, Boston, Massachusetts, United States; 3 Bedford VA Medical Center, Bedford, Massachusetts, United States

## Abstract

Nursing home safety climate in nursing homes reflects norms and attitudes about the safety of residents and is a key driver of safety. We investigated views of direct care staff and senior managers in 56 Department of Veterans Affairs nursing homes, because prior hospital studies reveal incongruent views of these two groups, which compromises quality of care. Each domain of the previously validated CLC Employee Survey of Attitudes about Resident Safety (CESARS) served as a dependent variable, with occupation as the major independent variable distinguishing senior managers (the reference group) from licensed nurses, nursing assistants, and clinicians/specialists. Mixed random effect models controlled for job tenure, work shift, ≤ 40 weekly work hours or more, as well as clustering effects (by VA hospitals, VA service networks, and geographic regions). We analyzed responses of 1316 direct care staff and senior managers, a 26% response rate. Senior managers were more favorable about their co-worker interactions regarding safety and their nursing home globally than each direct care staff group (small to moderate effects or ⅓ to ½ of a standard deviation) (p < 0.05). Direct care staff had comparable ratings on these two safety climate domains. We found incongruence between senior managers and direct care staff in their perceptions of their facility’s safety. Results imply that regular and open conversations between direct care staff and senior managers around safety may keep senior managers informed about frontline safety issues and direct care staff about high-level quality improvement initiatives, bringing them closer to a mutual understanding.

